# Research Note: Injurious pecking in fattening turkeys (*Meleagris gallopavo* f. dom.)—video analyses of triggering factors and behavioral sequences in small flocks of male turkeys

**DOI:** 10.1016/j.psj.2020.09.016

**Published:** 2020-09-13

**Authors:** T. Bartels, R.A. Stuhrmann, E.T. Krause, L. Schrader

**Affiliations:** Friedrich-Loeffler-Institut, Institute for Animal Welfare and Animal Husbandry, Celle, Germany

**Keywords:** fattening turkey, injurious pecking, feather pecking, eliciting factor, animal welfare

## Abstract

Injurious pecking is one of the main welfare issues in fattening turkey husbandry. Birds pecked by conspecifics can suffer from serious injuries that may even lead to the death of the victimized animals or require their culling. In the present study, the behavior of male turkeys was documented day and night using video recordings throughout the entire fattening period of 19 wk. Thus, when a turkey was found injured or dead in the barn, a retrospective analysis of video recordings was carried out to clarify the circumstances surrounding the death or injuries of the bird in the present study. In 3 fattening trials with a total of 1,620 male turkeys with intact beaks, 41 birds (2.5%) were found either seriously injured (n = 24) or dead (n = 17) in the barn as a result from conspecific pecking. The detailed evaluation of the video recordings showed that the onset of injurious pecking was mainly in the afternoon after the last daily visual controls of animals and that one third of the dead found animals died a natural death without any pecking incidents. The duration of injurious pecking directed against a certain conspecific was on average 794 min, ranging from 84 min to 1,437 min, that is up to almost an entire day. Pecking activities lasting more than 10 h were interrupted from the light regime between dusk and dawn but continued with the onset of light in the morning. Duration of injurious pecking events decreased with age (*P* = 0.031). If the victims laid down or were impaired in their mobility, they would be surrounded by up to 10 turkeys that would still be forcefully pecking at the occipital and neck area of the badly injured or moribund birds.

## Introduction

In fattening turkeys, especially in male turkeys, injurious pecking is one of the main welfare and health issues in conventional as well as organic turkey fattening. Profound injuries to the head integument and its appendages as well as to the underlying tissue due to pecking by conspecifics are the main consequences. In the worst case, turkeys suffer from serious injuries that can lead to the death of the victimized animal or require its culling by the farmer (Krautwald-Junghanns et al., 2011). Thus, this undesired behavioral pattern leads to reduced health and suffering in the late stages and is therefore relevant to animal welfare and also raises economic concerns as animals dying before slaughter cannot be marketed. The causes for the development of injurious pecking are considered as multifactorial (Dalton et al., 2013), similar to allopecking behaviors in laying hens (Kaukonen and Valros, 2019). In turkeys, inadequate housing and management conditions, feed composition, or breed and sex are considered to be potential factors contributing to injurious pecking (Dalton et al., 2013, 2018; Duggan et al., 2014; Erasmus, 2017; Ferrante et al., 2019), but detailed knowledge on the development of injurious pecking in turkey flocks is still lacking (Marchewka et al., 2013). This would be especially important as the abnormal behavior becomes only visible in practice when already bleeding injuries are apparent and, thus, fatal consequences are initiated. However, it is almost unknown how long it does take to that stage and how behavioral sequences look like before severe injuries occur. So far, often beak trimming is conducted to reduce the damage caused by injurious pecking in fattening turkey husbandry (Dalton et al., 2017). Owing to an increasing public discussion on noncurative surgical procedures, in the European Union, including Germany, beak trimming of turkeys shall be banned in the future (Kulke et al., 2016). Currently, it is difficult to keep non–beak-trimmed fattening turkeys under conventional conditions without a high proportion of injurious pecking (Kulke et al., 2016). Similar applies, albeit with lower prevalence, to organic turkey husbandry. In countries where beak trimming is forbidden, often light intensity in turkey housing is relatively low, without having substantial effects on injurious pecking as indicated by not substantially reduced mortality rates (Marchewka et al., 2019). To understand the underlying mechanisms of injurious pecking (Dalton et al., 2013), it will be helpful to learn more about the temporal course and durations of sequences of injurious pecking. Therefore, the aim of this study was to get further information on factors that can elicit injurious pecking in non–beak-trimmed male turkeys. To do so, retrospective evaluation of video recordings from pecking activities with fatal endings, that is severely injured animals that needed to be culled or found dead animals, was conducted to track the entire pecking sequence from the subtle first-pecking interaction, which led not yet to injuries and thus could not be detected during the visual controls twice a day, to severe visible injuries where intervention was then possible.

## Animals, materials, and methods

In 3 trials, 540 male day-old turkeys of the strain British United Turkeys 6 (Aviagen Group, Newbridge, UK) with intact beaks were allocated to 12 barn compartments (each 4.5 m × 4.0 m, with 45 turkeys per compartment ≙ 2.5 turkeys/m^2^, stocking density ≤ 53 kg/m^2^) littered with wood shavings. The compartments were separated from each other by solid walls, so that birds from adjacent compartments could not see each other. All turkeys were kept for 19 wk under common commercial conditions as per the German voluntary agreements for the husbandry of turkeys (VDP, 2013). Each compartment was equipped with 2 feeders (Imperator; Big Dutchman, Vechta, Germany) and 2 round drinkers (Jumbo-98; Big Dutchman, Vechta, Germany) that were accessible to the turkeys from all sides. The turkeys were fed with a standard 6-phase system diet (Fa. Agravis Raiffeisen AG, Münster, Germany). Each barn compartment was equipped with a pecking block (Vilovoss medium; Deutsche Vilomix Tierernährung GmbH, Neuenkirchen-Vörden, Germany) as environmental enrichment. Compartments were climate controlled and lightproof shielded against external light. Illumination was performed by light-emitting diode lamps (Telesto 8 W; LEDFactory B.V., Leeuwarden, The Netherlands) at an intensity of at least 20 lx. The lighting program was set to a 16L:8D light–dark cycle from day 7 on. The daily photoperiod lasted from 5:00 am to 9:00 pm and included a 20-min twilight phase in the mornings and evenings. Each compartment was equipped with 1 camera (VTC-E220IRP; Santec BW AG, Ahrensburg, Germany) located centrally at the compartment ceiling and equipped with wide-angle lenses (2.1 mm focal width, and 1/3-inch sensor size). The videos were recorded via a computer on hard disks (2 TB; Seagate Technology LLG, Cupertino). The behavior of the turkeys was recorded 24 h/d using digital video recordings throughout the entire fattening period. By video, the birds could not be identified individually.

All barn compartments were visually inspected at least twice a day, usually between 7:00 am and 9:00 am and between 1:00 pm and 3:00 pm to identify visible injured animals. Turkeys with minor injuries were medicated and transferred to a separate sick bay and kept there until the end of fattening.

In the rare case, a turkey had injuries that required its immediate culling for reason of animal welfare or was found dead in the barn compartment, a retrospective analysis of video recordings was carried out to investigate the circumstances of the death or injury of the animal. The period between the removal of the injured or dead turkey and the first contact between the subsequent victim and the turkey from which the initial injurious pecking originated was evaluated retrospectively. The initial contacts and much of the initial phase appear without injuries and include only behavioral interactions (see in the following) that are, thus, hard to spot at visual controls. The VLC media player was used for this analysis, and the videos were watched at an up to 8-fold speed. Observed behaviors are defined in Table 1. For statistical analysis, we calculated Spearman rank correlation tests between duration of injurious pecking and the age of the birds using R 4.0.0 (R Core Team, 2020).

**Table 1:** Definitions of behaviors.

Behavior pattern	Description
Agonistic behavior	A turkey is pecking another turkey. Mainly the back of the head of the recipient is targeted.
Chase	An injured turkey is pursued by one or more conspecifics when it attempts to escape
Copulation movements	After mounting the victim, the turkey performs reciprocating motions by lowering the wings, bracing the back feathers and bending the tail feathers downward
Mounting	A turkey climbs on the back of the moribund or already dead victim that lays sprawled out on the ground.
Severe feather pecking	A turkey is forcefully pecking another turkey on the plumage. This can lead to pulling and removing of individual feathers
Torment	One or more turkeys are physically oppressing another turkey. The victim is being surrounded and pecked on the occiput by its conspecifics.

## Results and discussion

In turkeys, allopecking can be divided into gentle or severe feather pecking of the body of a conspecific or aggressive pecking specifically directed toward its head and neck (Dalton et al., 2013). In contrast to gentle feather pecking, head pecking and severe feather pecking are classified as injurious pecking as these behaviors can cause tissue damage and mortality resulting in declines in both productivity and welfare (Krautwald-Junghanns et al., 2011; Dalton et al., 2013).

In total, 47 male turkeys (2.9%) were found seriously injured or dead in the barn during the 3 fattening trials. All severely injured but still-living turkeys (n = 24) had to be culled owing to an unfavorable prognosis. They showed deep injuries at the back of the head or the neck or both due to skin-penetrating blows by beaks, some of which extended to the skull or spine. Of the 23 turkeys found dead, 17 birds had significant injuries at the back of the head apparently inflicted by conspecifics. The other 6 turkeys found dead had no noticeable premortal injuries. Retrospective evaluation of video recordings showed that these birds spontaneously died without any identifiable external influence. Necropsy of these 6 birds showed signs of intestinal inflammations, swelling of the liver, and kidney damages. Thus, in total, 41 animals, which resembles 2.5% of all animals (n = 1,620) and accordingly 39.1% of the total losses (6.5%; n = 105) in the 3 trials, died as a result from conspecific injurious pecking. The remaining 64 losses were related to turkeys that were brought to a sick bay because of illness or accidental injuries and perished there or had been culled. Regarding death rates, there were no serious differences between the 3 trials (trial 1: n = 15; trial 2: n = 26; trial 3: n = 23).

**Figure 1:**
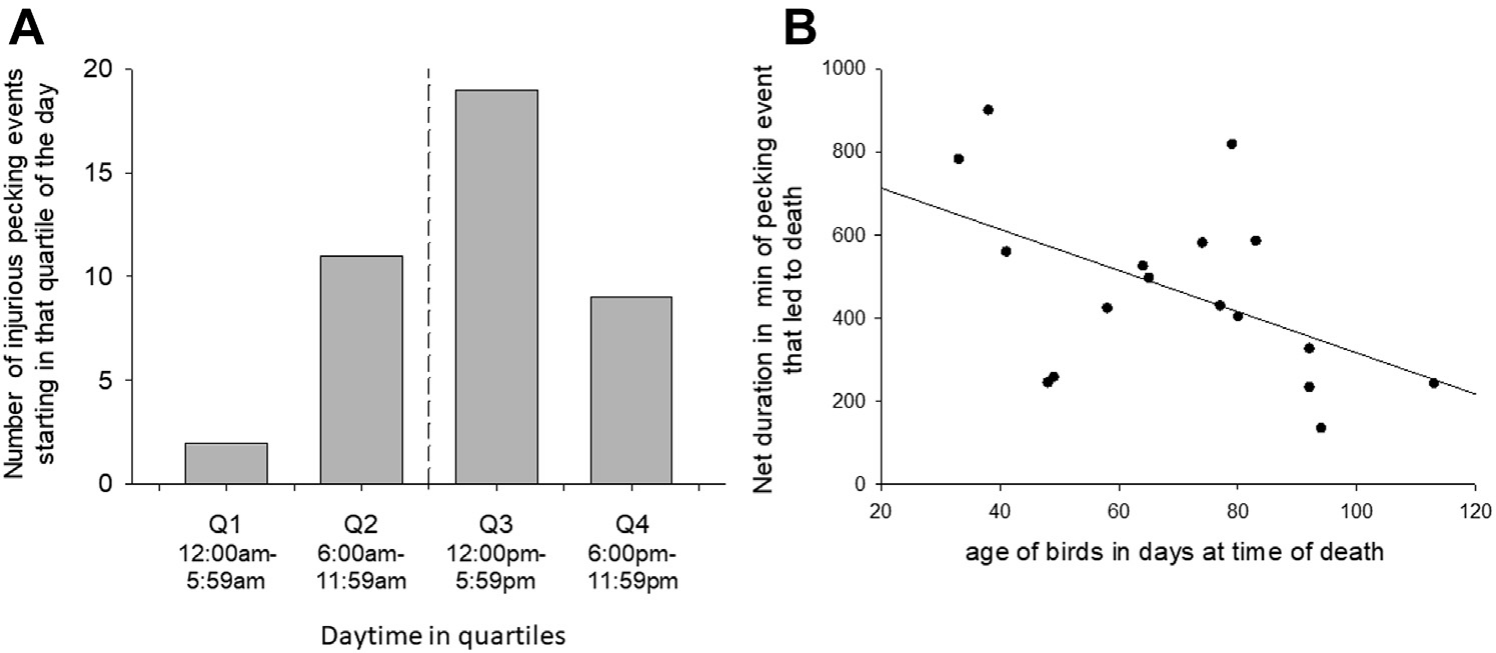
(A) Daytimes divided in quartiles in which the initial phase of injurious pecking events of male turkey started. (B) Age of male turkeys (in days) at which they perished in relation to the duration of the respective injurious pecking event (Spearman correlation, n = 17, r_S_ = -0.523, *P* = 0.031). (B) only animals were included where the full behavioral sequence of injurious pecking was available, i.e. the pecking ended fatal as in-time removal of the animal was not possible.

The evaluation of the video recordings showed that the initial phase (i.e., first agonistic interaction between subsequent victim and another turkey) for injurious pecking was mainly in the afternoon of the day (n = 28) (Table 2). Less often, the beginning of an injurious pecking was observed in the morning (n = 13) (Figure 1A). The duration from initial pecking directed against a conspecific to the culling or death of the victim was on average 794 min ± 54 min (SE), ranging from 84 min to 1,437 min. Pecking phases lasting more than 10 h were interrupted in the evening with the onset of twilight and only continued with the onset of lighting the next morning (Table 2). When excluding the dark phase of 7 h 20 min from the durations of pecking, the net duration of pecking phase was 450 min ± 33 min (SE), which, however, can be distributed on 2 subsequent day. Net duration of pecking phases did not correlate with the age of the birds, when considering all 41 cases with dead-found and culled birds (Spearman correlation, n = 41, r_S_ = -0.222, *P* = 0.161). However, in the culled turkeys (n = 24), we terminated the pecking phases for ethical reasons. Thus, the full behavioral sequence is only available from 17 animals found dead. Interestingly, for these turkeys, we found a negative relationship between age and net duration of pecking (Spearman correlation, n = 17, r_S_ = -0.523, *P* = 0.031; Figure 1B). With increasing age, the pecking phase resulting in dead victims decreases. This may indicate that either younger victims are more active, which is why they are more likely to escape or younger turkeys are still less motivated to perform injurious pecking. Probably with increasing age, beak strokes are also executed with greater force, which leads quickly to serious injuries for the victim. In 19 of the 41 cases, the injurious pecking developed either from plucking of the plumage (n = 3) or of the skin of the head and neck region (n = 16). Five of these turkeys had older, already scabbed skin wounds, which were repecked by conspecifics. One bird had a round skin injury with a diameter of approximately 2 cm on the occiput. Two birds showed an oblong skin lesion with a length of approximately 3–4 cm on the neck. Two turkeys had an injury of similar shape and size but one was on the shoulder and the other one on the flank. The etiology of these lesions (pecking or scratching by conspecifics, abrasion) remained open. Locomotion disorders such as lameness and/or disturbance of equilibrium were observed in 5 other birds that later became victims. Yet, 2 other turkeys appeared in poor condition, recognizable by a crouching posture and ruffled plumage. In another case, injurious pecking developed without noticeable cause from a general arousal within the flock. In 14 cases, injurious pecking followed after agonistic behavior between 2 male turkeys.

**Table 2:** Injurious pecking in fattening turkeys. Age of turkeys injured or killed by conspecifics, time frame and duration of injurious pecking, eliciting factors, and behavioral sequence as revealed by video analyses.

Trial	Case	Age [d]	Time frame			Observations			
			Start		Duration [h:min]	Initial phase	Main phase	Final phase	Ultimate result
			Date	Time					
1	1	41	19.12.2017	2:52 pm	16:40∗	Bird was pecked on the occiput	Torment, chase	Torment	Killed
	2	48	26.12.2017	7:58 pm	11:25∗	Bird was pecked on the occiput	Torment, chase	Torment, mounting	Killed
	3	64	11.01.2018	3:57 pm	16:06∗	Bird was pecked on an encrusted wound	Torment, chase	Torment	Killed
	4	65	12.01.2018	4:55 pm	15:37∗	Bird was pecked on the occiput	Torment, chase	Torment, mounting	Killed
	5	71	18.01.2018	9:23 am	5:01	Agonistic interaction between 2 males	Torment, chase	Torment, mounting	Culled
	6	72	19.01.2018	8:15 am	3:42	Agonistic interaction between 2 males	Torment, chase	Torment, mounting	Culled
	7	77	24.01.2018	5:11 pm	14:30∗	Agonistic interaction between 2 males	Torment, chase	Torment, mounting	Killed
	8	78	25.01.2018	2:53 pm	16:49∗	Agonistic interaction between 2 males	Torment, chase	Torment, mounting	Culled
	9	79	26.01.2018	12:47 pm	20:59∗	Bird was pecked on an encrusted wound	Torment, chase	Torment, mounting	Killed
	10	80	27.01.2018	7:32 pm	14:04∗	Agonistic interaction between 2 males	Torment, chase	Torment, mounting	Killed
	11	82	29.01.2018	5:16 pm	14:23∗	Agonistic interaction between 2 males	Torment, chase	Torment, mounting	Culled
	12	83	30.01.2018	2:29 pm	17:06∗	Agonistic interaction between 2 males	Torment, chase	Torment, mounting	Killed
	13	86	02.02.2018	6:32 pm	13:59∗	Bird was pecked on an encrusted wound	Torment, chase	Torment, mounting	Culled
	14	87	03.02.2018	5:06 am	3:26	Feather pecking	Torment, chase	Torment, mounting	Culled
	15	90	06.02.2018	7:24 pm	12:21∗	Agonistic interaction between 2 males	Torment, chase	Torment, mounting	Culled
	16	113	01.03.2018	8:35 pm	11:23∗	Bird was unable to rise	Torment	Torment, mounting	Killed
	17	120	08.03.2018	8:00 pm	11:14∗	Bird was unable to rise	Torment	Torment, mounting	Culled
2	18	38	22.12.2018	10:56 am	22:21∗	Feather pecking	Torment, chase	Torment	Killed
	19	49	02.01.2019	7:54 pm	11:38∗	Bird appeared in poor condition	Torment, chase	Torment, mounting	Killed
	20	58	11.01.2019	8:01 pm	14:24∗	Bird showed disturbance of equilibrium	Torment	Torment, mounting	Killed
	21	74	27.01.2019	6:58 pm	12:36∗	Bird was pecked on the occiput	Torment, chase	Torment, mounting	Culled
	22	74	27.01.2019	2:44 pm	17:02∗	Bird appeared in poor condition	Torment, chase	Torment, mounting	Killed
	23	81	03.02.2019	5:52 pm	13:50∗	Agonistic interaction between 2 males	Torment, chase	Torment, mounting	Culled
	24	82	04.02.2019	11:40 am	19:41∗	Bird was pecked on the occiput	Torment, chase	Torment, mounting	Culled
	25	92	14.02.2019	8:02 pm	11:14∗	Bird showed disturbance of equilibrium	Torment	Torment, mounting	Killed
	26	92	14.02.2019	9:41 am	5:27	Agonistic interaction between 2 males	Torment, chase	Torment, mounting	Killed
	27	94	16.02.2019	7:29 am	2:15	General excitement within the flock	Torment, chase	Torment, mounting	Killed
	28	94	16.02.2019	4:36 pm	16:34∗	Bird was pecked on the occiput	Torment, chase	Torment, mounting	Culled
	29	112	06.03.2019	7:39 am	23:57∗	Agonistic interaction between 2 males	Torment, chase	Torment, mounting	Culled
3	30	33	15.06.2019	1:57 pm	20:23∗	Bird was pecked on the occiput	Torment, chase	Torment	Killed
	31	45	27.06.2019	4:44 pm	13:29∗	Bird was pecked on the occiput	Torment, chase	Torment	Culled
	32	48	30.06.2019	12:20 pm	17:46∗	Bird was pecked on an encrusted wound	Torment, chase	Torment	Culled
	33	52	04.07.2019	2:09 pm	16:00∗	Feather pecking	Torment, chase	Torment, mounting	Culled
	34	56	08.07.2019	7:32 am	6:48	Bird was pecked on an encrusted wound	Torment, chase	Torment, mounting	Culled
	35	94	15.08.2019	7:23 am	4:58	Agonistic interaction between 2 males	Torment, chase	Torment, mounting	Culled
	36	107	28.08.2019	11:48 am	19:13∗	Bird was pecked on the occiput	Torment, chase	Torment, mounting	Culled
	37	109	30.08.2019	5:00 am	4:30	Bird was unable to rise	Torment	Torment, mounting	Culled
	38	111	01.09.2019	6:55 am	1:24	Agonistic interaction between 2 males	Torment, chase	Torment, mounting	Culled
	39	114	04.09.2019	1:15 pm	17:40∗	Agonistic interaction between 2 males	Torment, chase	Torment, mounting	Culled
	40	121	11.09.2019	3:30 pm	15:17∗	Bird was pecked on the occiput	Torment, chase	Torment, mounting	Culled
	41	124	14.09.2019	5:17 pm	15:11∗	Bird was pecked on the occiput	Torment, chase	Torment, mounting	Culled

Initial phase: first agonistic interaction between subsequent victim and another turkey, subordinated bird is chased by the dominant turkey; main phase: victim is surrounded by numerous conspecifics, followed by pecking and chasing of the victim by a changing number of turkeys; final phase: victim surrenders, stops attempts to escape, and submits without reaction to further injurious pecking by conspecifics; culled: turkey had to be culled because of the severity of the injuries in accordance with animal welfare legislation; killed: turkey was killed by conspecifics through injurious pecking.

∗ Including a dark period (7 h 20 min) and 2 twilight periods with a duration of 20 min each from 5:00–5:20 am and 8:40–9:00 pm.

Losses from injuries that developed from severe feather pecking first occurred in the fifth week of life (Table 2). First, losses due to agonistic interaction between 2 males occurred in the 11th week of life. In fattening turkeys, the onset of injurious pecking directed against conspecifics falls into the stage of behavioral development in which both male and female turkeys in the wild also begin to become aggressive toward their siblings of the same sex. In wild turkeys, establishment of a social order within the family groups starts in the eighth week of life (Healy, 1992) and follows a certain pattern. They usually begin with threatening gestures. The turkeys display an upright posture with an erected neck and feathers, and the wings are slightly bent from the body and hanging down. This display is accompanied by characteristic vocalizations. When fighting occurs, the turkeys jostle each other, flap their wings toward the head of the opponent, and try to hit him with their beaks. The winner of a fight will usually follow/chase the loser for a while and will continue to peck him. In wild turkeys, these fights are usually of short durations and never lead to serious injury (Healy, 1992) as birds can spatially avoid each other. This leads to the creation of a stable hierarchy within the sympaedium, which is a prerequisite for cooperative courtship especially in male wild turkeys (Krakauer, 2005).

In contrast to wild turkeys, where these fights occur within small family groups, fattening turkeys are kept in large numbers on a limited area. In addition, stable social relationships that require individual identification of conspecifics cannot be established, neither in flocks of several thousand turkeys (Marchewka et al, 2013) nor in much smaller groups of about 30 individuals (Buchwalder, and Huber-Eicher, 2005). Within the scope of the present study, the retrospective analysis of the video recordings showed that agonistic conflicts between 2 members of a flock attracted the attention of others. The following observations of certain single events are estimated as potential representative by us and might be helpful for future studies on injurious pecking in turkeys. If the defeated turkey broke off the fight, he would not only be pursued by the opponent but often also by other turkeys that were not initially involved. In the beginning, we observed that the chased animal occasionally turned around to fend off the pursuers. During the chase, the pursuers tried again and again to peck at the back of the victims' head. This regularly led to bleeding injuries at the back of head and neck, that is the first potential signs when these animals can be identified and taken to a sickbay during visual controls. The chases could slow down in between but resumed promptly with more pecking.

In the main phase of an agonistic interaction, usually indicated by pecking and chasing, an increasing number of turkeys joined the chases. The victim continued trying to escape. To avoid being pecked, we observed that the chased animal tried to hide its head either in a corner of the pen or under a barn equipment (feeding trough, drinking trough, and so on). Sometimes, the bird also put its head under the body of a conspecific. Within the flock, a group dynamic developed toward the victim. In some cases, the victim was surrounded by up to 30 animals, with the entire group usually moving along a stable wall. Many animals made an excited impression, which was expressed in increasing motion activity, sometimes supplemented by flapping of wings. If the victim remained calm and motionless, the turkeys would quickly loose interest, and there were short periods of time in which the victim was not being tormented and could rest. However, once the victim moved minimally again, this often immediately attracted the attention of conspecifics. When the victims tried to escape again, the event was repeated. If the victims laid down or were impaired in their mobility, they would be surrounded by up to 10 turkeys who simultaneously pecked again at skin injuries that the defeated bird had meanwhile received at the back of the head. During the final phase, the victims surrendered and submitted without any reaction to the further injurious pecking of conspecifics at the occipital and neck area. This behavior also continued after the victims had died.

In the final phase, it was noticeable in 39 analyzed cases that, beginning in the eighth week of life, male turkeys in the immediate vicinity displayed behavior on the moribund or already dead turkeys that can be interpreted as copulation movements, that is the performing turkeys climbed onto the back of the moribund or already dead turkeys. Afterward, reciprocating motions were performed by lowering the wings, bracing the back feathers, and bending the tail feathers downward. This behavior led to further injuries on the back of the victim. A victim often laid sprawled out on the ground. That may trigger copulation because this position is also shown by mature turkey hens during pairing (Healy, 1992). In case 3, the bird died lying on its back. Here, no mounting attempts and reciprocating motions were performed by conspecifics.

The present study confirms previous findings that in small turkey flocks injurious pecking especially on the head and neck region often has its origin in agonistic interactions in the context of ranking fights (Dalton et al., 2013; Ferrante et al., 2019). Further studies are to be carried out to check whether the described behaviors also occur in large turkey flocks of several thousand individuals in a comparable form. A better understanding of the triggering factors and behavioral processes may help to develop strategies (e.g., concerning illumination management, environmental enrichment, or flock management), to reduce the occurrence of injurious pecking and to identify it before the occurrence of injuries. This can lead to a significant improvement of animal welfare in fattening turkey husbandry.

## Supplementary data

Authorship Statement

## References

[bib1] Buchwalder T., Huber-Eicher B. (2005). Effect of group size on aggressive reactions to an introduced conspecific in groups of domestic turkeys (*Meleagris gallopavo*). Appl. Anim. Behav. Sci..

[bib2] Dalton H.A., Wood B.J., Torrey S. (2013). Injurious pecking in domestic turkeys: development, causes, and potential solutions. World's Poult. Sci. J..

[bib3] Dalton H.A., Wood B.J., Widowski T.M., Guerin M.T., Torrey S. (2017). An analysis of beak shape variation in two ages of domestic turkeys (*Meleagris gallopavo*) using landmark-based geometric morphometrics. PLoS One.

[bib4] Dalton H.A., Wood B.J., Widowski T.M., Guerind M.T., Torrey S. (2018). Comparing the behavioural organization of head pecking, severe feather pecking, and gentle feather pecking in domestic turkeys. Appl. Anim. Behav. Sci..

[bib6] Duggan G., Widowski T., Quinton M., Torrey S. (2014). The development of injurious pecking in a commercial Turkey facility. J. Appl. Poult. Res..

[bib7] Erasmus M.A. (2017). A review of the effects of stocking density on Turkey behavior, welfare, and productivity. Poult. Sci..

[bib8] Ferrante V., Lolli S., Ferrari L., Watanabe T.T.N., Tremolada C., Marchewka J., Estevez I. (2019). Differences in prevalence of welfare indicators in male and female Turkey flocks (*Meleagris gallopavo*). Poult. Sci..

[bib9] Healy W.M., Dickson J.G. (1992). Behavior. The Wild turkey. Biology and Management.

[bib10] Kaukonen E., Valros A. (2019). Feather pecking and cannibalism in non-beak-trimmed laying hen flocks – Farmers’ perspectives. Animals.

[bib11] Krakauer A.H. (2005). Kin selection and cooperative courtship in wild turkeys. Nature.

[bib12] Krautwald-Junghanns M.-E., Ellerich R., Mitterer-Istyagin H., Ludewig M., Fehlhaber K., Schuster E., Berk J., Dressel A., Petermann S., Kruse W., Noack U., Albrecht K., Bartels T. (2011). Examination of the prevalence of skin injuries in debeaked fattened turkeys. Berl. Munch. Tierarztl. Wochenschr..

[bib13] Kulke K., Spindler B., Kemper N. (2016). A waiver of beak-trimming in turkeys – current situation in Germany. Züchtungskunde.

[bib14] Marchewka J., Watanabe T.T.N., Ferrante V., Estevez I. (2013). Review of the social and environmental factors affecting the behavior and welfare of turkeys (*Meleagris gallopavo*). Poult. Sci..

[bib15] Marchewka J., Vasdal G., Moe R.O. (2019). Identifying welfare issues in Turkey hen and tom flocks applying the transect walk method. Poult. Sci..

[bib16] R Core Team (2020). R: A Language and Environment for Statistical Computing.

[bib17] VDP (2013). Voluntary agreement on turkey husbandry guidelines (Bundeseinheitliche Eckwerte für eine freiwillige Vereinbarung zur Haltung von Mastputen).

