# Supplementary material for: Research Note: Injurious pecking in fattening turkeys (Meleagris gallopavo f. dom.)—video analyses of triggering factors and behavioral sequences in small flocks of male turkeys
Source: Poult Sci. 2020 Sep 13;99(12):6326–31. doi: 10.1016/j.psj.2020.09.016 (PMC7704993; doi:10.1016/j.psj.2020.09.016)
Supplement: Authorship Statement [file mmc1.docx]

Authorship Statement

The idea for the study was conceived by Thomas Bartels and Lars Schrader. The retrospective analysis of video recordings was carried out by Rebecca A. Stuhrmann. The data were analyzed by E. Tobias Krause. The article was written by Thomas Bartels, Rebecca A. Stuhrmann, and E. Tobias Krause and approved by all authors.
